# Graphics Processing Unit-Accelerated Code for Computing Second-Order Wiener Kernels and Spike-Triggered Covariance

**DOI:** 10.1371/journal.pone.0169842

**Published:** 2017-01-09

**Authors:** Omer Mano, Damon A. Clark

**Affiliations:** 1 Department of Molecular, Cellular, and Developmental Biology, Yale University, New Haven, Connecticut, United States of America; 2 Department of Physics, Yale University, New Haven, Connecticut, United States of America; 3 Interdepartmental Neuroscience Program, Yale University, New Haven, Connecticut, United States of America; Centre de Regulacio Genomica, SPAIN

## Abstract

Sensory neuroscience seeks to understand and predict how sensory neurons respond to stimuli. Nonlinear components of neural responses are frequently characterized by the second-order Wiener kernel and the closely-related spike-triggered covariance (STC). Recent advances in data acquisition have made it increasingly common and computationally intensive to compute second-order Wiener kernels/STC matrices. In order to speed up this sort of analysis, we developed a graphics processing unit (GPU)-accelerated module that computes the second-order Wiener kernel of a system’s response to a stimulus. The generated kernel can be easily transformed for use in standard STC analyses. Our code speeds up such analyses by factors of over 100 relative to current methods that utilize central processing units (CPUs). It works on any modern GPU and may be integrated into many data analysis workflows. This module accelerates data analysis so that more time can be spent exploring parameter space and interpreting data.

## Introduction

An important goal in neuroscience is to understand how stimuli influence the activity of neurons. One way to characterize this influence is with linear kernels, which describe how neural responses depend linearly on past and present stimuli. These kernels do not tell the entire story, since neural responses are frequently nonlinear. One solution is to combine linear kernels with static nonlinearities to account for neural nonlinearities [1]. An alternative is to directly determine how the response depends on different correlations in the input, an approach known as nonlinear systems identification [2]. In this approach, the nonlinear response can be approximated to low order by the second-order Wiener kernel of the system [2, 3], which is closely related to spike-triggered covariance (STC) methods [4–9]. The shape of the Wiener kernel is informative about which correlations elicit responses, and its eigenvectors span the space of potential linear feature detectors that could be combined nonlinearly to generate the response [5, 10]. These second-order analyses have been used to characterize photoreceptors [11, 12], retinal ganglion cells [6, 13–17], visual direction-selective cells in cortex [18–20] and in insects [10, 21, 22], direction-selective behavior in insects [22, 23], somatosensory neurons [24–26], olfactory receptor neurons [27] and subsequent olfactory processing neurons [28], auditory neurons [29, 30], electroretinograms [31, 32], and functional magnetic resonance imaging [33, 34]. These studies used second-order kernels to gain insights into timescales and lengthscales of motion computations, relevant spatiotemporal features for retinal and cortical visual neurons, and distinct ON and OFF pathway inputs.

The computational load for analyzing data in neuroscience is growing heavier, largely because data sets are increasing in size as the number of recorded cells, the sampling rate of cells, and the duration of recordings all increase. Multi-electrode arrays allow extracellular recordings of hundreds of cells [35, 36], while one- and two-photon imaging techniques now also allow hundreds of cells to be recorded [37, 38]. Genetically encoded indicators of activity are becoming faster [39–43] and imaging techniques are acquiring data with increasingly high sampling rates [42]. Covering relevant timescales at faster sampling rates increases the load for analysis.

Calculating second-order filters can require trillions of mathematical operations per filter. To date, second-order kernels have been extracted using standard libraries on central processing units (CPUs) [44]. Graphics processing units (GPUs) can perform some types of computations far more efficiently than CPUs [45]. In our own research, we were computing and assessing the significance of second-order Wiener kernels for thousands of 2-photon calcium imaging traces, each with more than 10,000 samples in time [22]. In order to speed up these computations, we wrote code to extract response-weighted stimulus covariance matrices (Wiener kernels) using GPUs.

## Results

**Fig 1:**
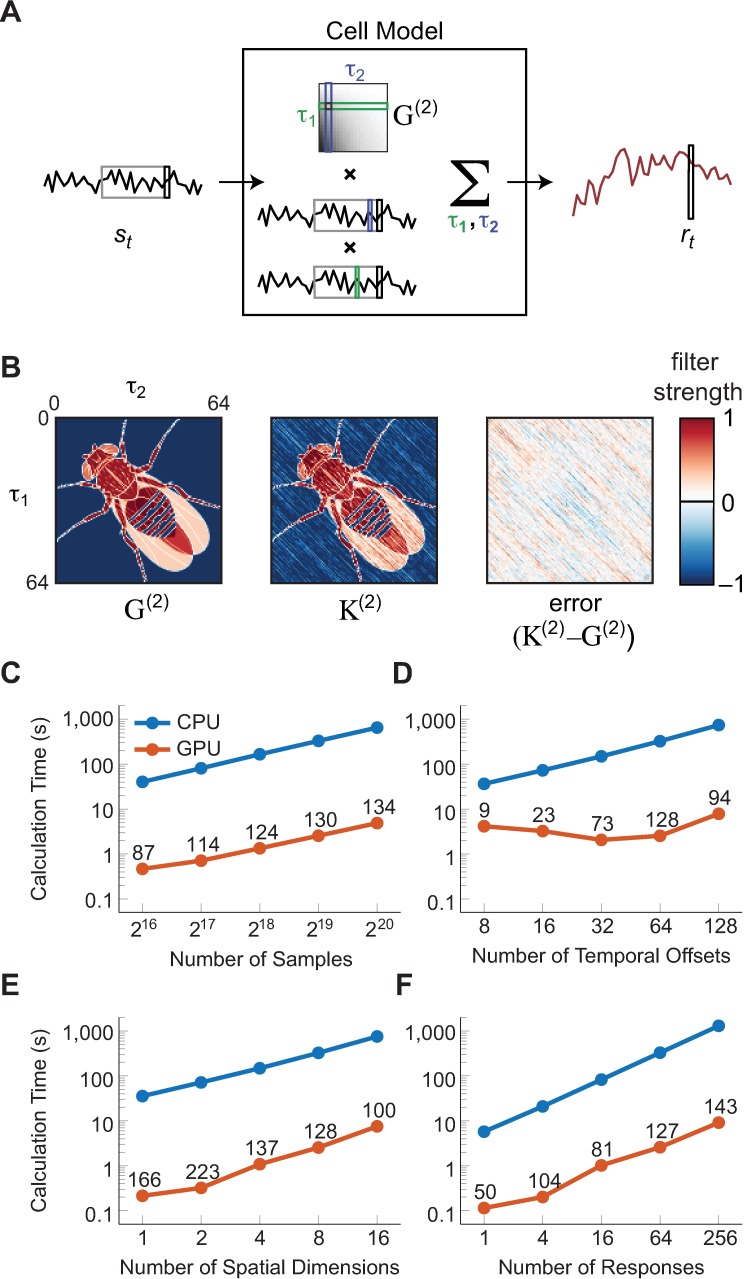
Verified algorithm and degree of speed up. (A) The second order component of a cell’s response is modeled as a linear weighting of pairwise products in the stimulus. The linear weighting matrix is the Volterra kernel, *G*^(2)^. (B) The GPU code correctly extracts the Wiener kernel from the stimulus-response pair. We simulated a model cell that used a Volterra kernel *G*^(2)^ to respond to a Gaussian input with unit variance. We then used the GPU code to estimate the Weiner kernel *K*^(2)^. Differences between the actual and estimated kernel go to 0 as the number of stimulus-response samples increases. In this case, the Wiener kernel equals the Volterra kernel because the response does not depend on higher order functions of the stimulus. (C-F) Performance of the GPU-enabled module versus the reference CPU implementation. Unless otherwise stated for each simulation parameter sweep, the dataset included 64 responses of 2^19^ samples, with inputs of 8 spatial dimensions each. The code extracted filters with 64 temporal offsets. All calculation times are given in seconds; code was run on hardware as described in Methods. Numbers above the GPU datapoints indicate the factor speedup of the GPU module relative to the CPU module. (C) Calculation time comparison for a sweep of the number of samples of each response. (D) Calculation time comparison for a sweep of the number of temporal offsets. (E) Calculation time comparison for a sweep of the number of spatial dimensions. (F) Calculation time comparison for a sweep of the number of responses (e.g. recorded cells in an imaging dataset).

Our code uses the computer’s GPU to compute second-order Wiener kernels (see Methods for the formula and scaling of the kernel). To test our GPU-accelerated code, we generated a synthetic dataset that creates a response from a second-order Volterra kernel acting on a discrete, Gaussian, uncorrelated input (Fig 1A). We then computed the second-order Wiener kernel for this stimulus-response set (Fig 1B). The Wiener kernel is an unbiased estimate of the Volterra kernel when the system has no higher-order responses and when the response is mean-subtracted. The error in the kernel estimate can be attributed to the finite stimulus presentation. This simulated kernel has very high rank for illustrative purposes, but a real extracted kernel is likely to be much lower rank [5, 6].

To quantify the advantages of the GPU-based algorithm, we compared its performance to that of a CPU-based algorithm on synthetic datasets with different properties. We first varied the duration of the stimulus, expressed in the number of samples. We found that over a wide range of samples, the GPU-algorithm beat the CPU-algorithm by a factor of over 100 (Fig 1C). The speedup increases as the number of samples increases because the GPU module’s initialization time, while shorter in absolute terms than the CPU implementation’s initialization time, makes up a larger proportion of the total runtime. As the number of samples increases, this initialization time becomes a smaller fraction of the total time.

The number of operations needed to compute the second-order kernel scales with the square of the number lags in the filter. As the number of lags increases, the GPU-algorithm speeds up relative to the CPU-algorithm (Fig 1D). The optimal filter size for the GPU-algorithm on the tested GPU is 64 lags, due to how cores are grouped in this particular GPU (different GPUs may have different optimal lags). Above 64 lags, the GPU-algorithm’s relative speed up remains roughly constant. When the user requests fewer than 64 lags, fewer GPU cores are allocated to the task, so that the code slows somewhat (in terms of calculations per second) when fewer than 64 lags are requested.

As the number of dimensions of the stimulus increases, the number of elements in the second-order kernel increases quadratically. The GPU-algorithm remains about 100 times faster than the CPU-algorithm as the number of dimensions increases (Fig 1E), but the gains are greater at lower numbers of dimensions. The CPU implementation becomes relatively more efficient as the number of spatial dimensions increases, but does not catch up to the GPU implementation.

New methods of data acquisition can simultaneously acquire many neural responses to a single stimulus. In the GPU-algorithm implementation, the speed up relative to the CPU increases as the number of responses increases (Fig 1F). This is because computing the Wiener kernels of fewer than 64 responses typically does not contain enough work to keep all the cores of a GPU busy.

**Fig 2:**
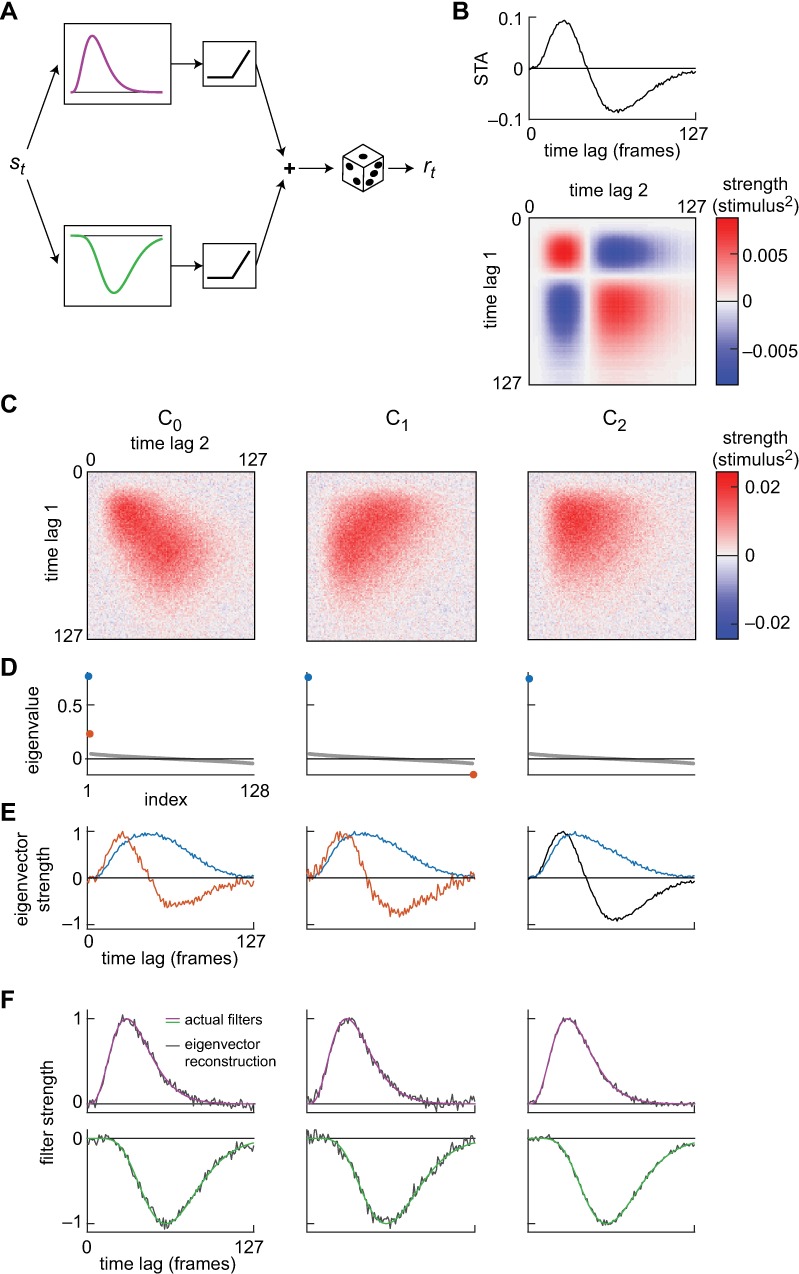
Demonstration of input filter extraction from a model spiking cell. (A) A model spiking neuron. Two linear filters act on the input, are rectified, and then summed. This determines the probability of firing, which is modeled as a Poisson process. Gaussian random inputs were fed into the model and resulting spikes computed with a mean rate of 0.0045 per time bin. (B) We extracted a spike triggered average from the responses (top). The extracted filter is a mix of the two input filters, and does not accurately represent the underlying linear processing. The outer product of the spike triggered average (bottom) will be used to compute forms of the STC matrix. (C) Three forms of the spike triggered covariance (STC) matrix: raw (*C*_*0*_), STA subtracted (*C*_*1*_), and with the STA projected out (*C*_*2*_). See Methods for details. (D) The eigenspectrum of each STC matrix, with significant eigenvalues highlighted. (E) Eigenvectors of each STC matrix that correspond to significant eigenvalues. In the case of *C*_*2*_, the STA serves as the second filter. (F) Comparison of the actual input filters (purple and green) with linear combinations of the extracted filters (grey); the eigenvectors span the space of linear input filters.

Perhaps the most common application of the kernels we compute is in spike triggered covariance (STC) analysis, when these kernels are used to determine the space of linear filters that contribute to a cell’s activity. In order to demonstrate how our algorithm can be used for this purpose, we simulated a spiking neuron whose firing rate is the sum of two linear-nonlinear models (Fig 2A), reminiscent of ON and OFF inputs to cells that have been similarly analyzed [46]. We presented the model cell with Gaussian random inputs, and simulated the resulting spike train as a Poisson spiking process. The spike-triggered average (STA) does not accurately represent either one of the input filters, and instead mixes the two input filters together (Fig 2B). In order to reconstitute the input filters, we first extracted the response-weighted stimulus covariance using the algorithm presented here. (This is proportional to the Wiener kernel; see Methods.) We then performed computationally trivial matrix operations to convert the resulting kernel into the most commonly used forms of the spike triggered covariance matrix (Fig 2C, see Methods for derivation and conversion instructions). We first computed the raw response-weighted covariance matrix (Fig 2C, *left*). We computed two other manipulations of this matrix popular for STC analysis, in which the STA is either subtracted (Fig 2C, *middle*, [6]) or projected out of the matrix (Fig 2C, *right*, [18]). The Methods section describes these operations in detail.

In each of the three cases, we calculated the eigenspectrum of the STC matrix and selected the eigenvectors with significant eigenvalues (Fig 2D and 2E). When the STA is projected out of the STC matrix, only one significant eigenvector remains; the STA itself is used as an additional vector for further analysis. The eigenvectors of these matrices span the space of possible linear filters in the model cell. The actual input vectors used in the model cell can be reconstituted from linear combinations of the significant eigenvectors (Fig 2F).

## Discussion

This code speeds up second-order Wiener kernel computations and STC analyses. The speed-up occurs over a wide range of data parameters (Fig 1), and is especially useful for large datasets that are sampled frequently in time and for experiments in which many neurons are simultaneously measured. The computational speed is increased by using the GPU to quickly perform low-level computations; this contributes to a trend of using GPU-processing to speed up computation-intensive tasks like machine learning and image analysis [47–49].

The second-order Wiener kernel is closely related to spike triggered covariance analysis (STC) [4]. The Wiener kernel allows for continuous and negative inputs, such as voltage, current, and light intensity. To use this code with spikes in computing an STC matrix, the response can simply code the number of spikes in each bin. In some implementations of STC, the spike triggered average (STA) is subtracted from the stimulus before computing covariance [6]. Other implementations of STC have subtracted off the component of the stimulus in the direction of the STA before computing the kernel [18]. To compute the STC in both these cases, this code can be used and its output can be transformed appropriately using the STA (see Methods). Fig 2 shows how the different methods might be applied to a simple example case, in each case first computing a response-weighted stimulus covariance matrix with the GPU code, then transforming it into the more familiar STC matrices that appear in the literature [6, 18].

It can be difficult to assess the statistical significance of Wiener kernels/STC matrices, since noise in the filter tends to be correlated across many elements. One method for significance estimation is to decorrelate the stimulus and response, time shifting them relative to one another, to obtain a filter estimate that would occur if the stimulus and response were unrelated [8]. This preserves the correlations in both the response and stimulus, so it has an appropriate noise distribution for a null hypothesis. To compare a measured filter to the decorrelated null set of filters, one needs to compute thousands or tens of thousands of decorrelated filters. If each filter set takes 100 seconds to compute, as it can with CPU code, this can be prohibitively difficult, taking approximately 10 days to compute a full null set of 10,000 filters. With the GPU acceleration, this can be narrowed to a few hours of computation. Thus, this code makes this method of significance testing far easier for Wiener kernels and STC analysis.

Overall, the increased analysis speed of this tool allows researchers to spend more time devising analyses and interpreting results and less time waiting for computations to finish. The time-savings encourages broader exploration of large neural datasets.

## Methods

We wrote code to compute the response-weighted stimulus covariance, *C*, from a paired stimulus and response:

$$C_{ij}=\frac{1}{T-\tau }\sum_{t=\tau +1}^{T}r_{t}s_{t-i}s_{t-j}$$

where *r*_*t*_ is the response at time *t*, *s*_*t*′_ is the stimulus at time *t*', *T* is the total length of the stimulus-response sampling, and *i* and *j* represent lags in the filter ranging from 0 to *τ*. When the stimulus is multidimensional and can be divided into separate time series, for instance representing multiple pixels on a screen, *C* is organized block-wise for each stimulus pairing, as described in the code documentation. This weighted average of the stimulus covariance is proportional to the second-order Wiener kernel when ⟨*r*_*t*_⟩ = 0. The second-order Wiener kernel, *K*^(2)^, is

$$K^{\left(2\right)}=\frac{1}{2\sigma^{4}\Delta t^{2}}C$$

where *σ* is the standard deviation of the input stimulus and Δ*t* is the sampling interval [2].

To see how the matrix *C*, computed by this algorithm, relates to different spike-triggered methods, we must define a few more quantities (for a more detailed analysis, see also [4]). First, in keeping with convention of the spike-triggered literature, we set *r*_*t*_ to be the integer number of spikes in the time window around time *t*, and we do not set the mean response to 0. Then, the spike triggered average (STA), **a**, is

$$a=\frac{1}{n_{r}}\sum_{t}r_{t}s_{t}$$

where **s**_*t*_ is a column vector of the stimulus preceding time *t*, and $n_{r}=\sum_{t}r_{t}$ is the total number of spikes. Because the response is non-zero in this case, we must also compute the stimulus covariance. That stimulus covariance may be computed by the GPU algorithm by setting all responses to 1, so that $S_{ij}=\frac{1}{T-\tau }\sum_{t=\tau +1}^{T}s_{t-i}s_{t-j}$, or $S=\frac{1}{T-\tau }\sum_{t=\tau +1}^{T}s_{t}s_{t}^{T}$. Then the raw spike triggered covariance (STC) matrix, *C*_0_, is just

$$C_{0}=\frac{1}{n_{r}}\sum_{t}r_{t}s_{t}s_{t}^{T}-S=\frac{T-\tau }{n_{r}}C-S$$

which is the response-weighted covariance we found above, *C*, scaled by the mean firing rate, with the basal stimulus covariance subtracted off [10]. Here and throughout, we assume that the number of samples for estimating the covariance is much larger than 1, so that the denominator may be written as *n*_*r*_ rather than *n*_*r*_−1 without appreciable error.

In some versions of STC, the STA is subtracted before computing the covariance [6]. In such a version, *C*_1_ is computed as

$$C_{1}=\frac{1}{n_{r}}\sum_{t}r_{t}(s_{t}-a){\left(s_{t}-a\right)}^{T}-S$$

This version reduces to *C*_1_ = *C*_0_−*A*, where *A* = **aa**^*T*^ is the outer product of the STA [4]. This means that the matrix computed by this algorithm, *C*, can be transformed into the form of *C*_1_ by simple matrix operations.

An alternative method computes the STC matrix by first removing the component of each stimulus vector in the direction of the STA [18]. This means subtracting a term **b**_*t*_ = **a**(**a**^*T*^**s**_*t*_)/(**a**^*T*^**a**) = *A*'**s**_*t*_ from each stimulus vector, and results in an STC matrix *C*_2_ defined as:

$$\begin{array}{l} C_{2}=\frac{1}{n_{r}}\sum_{t}r_{t}(s_{t}-b_{t}){\left(s_{t}-b_{t}\right)}^{T}-\frac{1}{T-\tau }\sum_{t}(s_{t}-b_{t}){\left(s_{t}-b_{t}\right)}^{T}\\ C_{2}=\frac{1}{n_{r}}\sum_{t}r_{t}(I-A')s_{t}s_{t}^{T}{\left(I-A'\right)}^{T}-\frac{1}{T-\tau }\sum_{t}(I-A')s_{t}s_{t}^{T}{\left(I-A'\right)}^{T} \end{array}$$

where *I* is the identity matrix and $A'=\frac{aa^{T}}{a^{T}a}$ is the outer product of the normalized STA. In the equations above, the stimulus has the projection subtracted before computing covariance, in the case of both the response-weighted covariance (first term) and the unweighted covariance (second term). This equation simplifies to *C*_2_ = (*I*−*A*')*C*_0_(*I*−*A*')^*T*^. Thus, in this case, too, the kernel *C*_0_ can be computed and transformed into the desired form with matrix multiplications.

GPUs achieve very high computational throughput by incorporating thousands of relatively slow “cores” in contrast to CPUs which contain a small number of relatively fast cores. GPUs can attain higher performance than CPUs when each core can perform an independent computation. Because each element *C*_*ij*_ can be computed independently, the Wiener kernel computation maps well to this architecture. Our module has also been tuned to remove bottlenecks in GPU computation (especially in the domain of memory accesses). Overall, we are able to achieve speeds which are very close to the considerable peak throughput of modern GPUs.

This code is available as a repository on Github:

https://github.com/ClarkLabCode/GPUFilterExtraction and is available for use under a GPL license. The code is written in C++ and OpenCL and will work for most modern GPUs (they must be compatible with OpenCL). We provide code that integrates it into Matlab, and it can be substituted into STC-packages like iSTAC [44]. Integration with R or Python is possible through each language’s C bindings. We have also included the code used to generate Figs 1 and 2.

We tested the GPU-accelerated code against standard existing code for second-order Wiener kernel computation (iSTAC [44]), which takes advantage of optimized linear algebra libraries to accelerate computations (Fig 1C–1F). For comparisons, the CPU and GPU code was run on the same machine with an Intel Core i7 6700K (4.0 Ghz) processor, an AMD Fury X GPU, and 32 GB of DDR4 RAM. The factors of speed up will be different using different CPUs and GPUs, but these are each reasonable high-end pieces of hardware for comparison.
